# NMR-Based Plasma Metabolomics at Set Intervals in Newborn Dairy Calves with Severe Sepsis

**DOI:** 10.1155/2018/8016510

**Published:** 2018-03-21

**Authors:** Abdullah Basoglu, Ismail Sen, Gaia Meoni, Leonardo Tenori, Amir Naseri

**Affiliations:** ^1^Department of Internal Medicine, Faculty of Veterinary Medicine, Selçuk University, Aleaddin Keykubat Campus 42003 Konya, Turkey; ^2^Magnetic Resonance Center (CERM), University of Florence, Via Luigi Sacconi 6, 50019 Sesto Fiorentino, Italy; ^3^Department of Experimental and Clinical Medicine, University of Florence, Largo Brambilla 3, 50134 Florence, Italy

## Abstract

The aim of this first study was to reveal the new potential biomarkers by a metabolomics approach in severe septic calves. Sepsis is a common cause of morbidity and mortality in newborn dairy calves. The main challenges with the use of biomarkers of sepsis in domestic animals are their availability, cost, and time required to obtain a result. Metabolomics may offer the potential to identify biomarkers that define calf sepsis in terms of combined clinical, physiological, and pathobiological abnormalities. To our knowledge, this is the first study presenting an NMR- (nuclear magnetic resonance-) based plasma metabolomics at set intervals in neonatal septic calves. Twenty neonatal dairy calves with severe sepsis and ten healthy calves were used. Hematological and biochemical health profiles were gathered in plasma samples at set intervals. Similarly, NMR spectra were acquired. All diseased animals (except one) died after 72 hours. Clinical and laboratory results were in accordance with those of severe septic animals. Multivariate analysis on NMR plasma spectra proved to be an excellent tool for faster identification of calves with severe sepsis from healthy animals. The NMR-based metabolomic profile may contribute to the better understanding of severe sepsis in newborn calves.

## 1. Introduction

The mortality risk of live-born newborn calves, 1 month of age, has been reported to range from 15 to 30%. The majority of deaths are attributable to infectious diseases; diarrhea, pneumonia, and sepsis are the most common. Sepsis is an acute systemic inflammatory response of the body to microbial infection and a life-threatening condition associated with multiple-organ failure [1]. Newborn sepsis is the third most common cause of calf mortality in the United States (behind diarrhea and respiratory disease) and occurs most commonly in calves associated with failure of passive transfer. The early signs of sepsis in newborn calves are vague and nonspecific and are often indistinguishable from signs of noninfectious diseases or those of focal infections such as diarrhea. Positive blood cultures are required for a definitive antemortem diagnosis of sepsis, but results are not reported for 48–72 hours, and false-negative culture findings are common. The high mortality of sepsis can be seen as an indication of insufficient laboratory diagnostics [2]. Unfortunately, despite many advances, the majority of laboratory investigations are not sufficiently sepsis specific. The laboratory diagnosis of sepsis is thus a mosaic of different technological and methodological approaches which are vital to the subsequent integration of the clinical picture and outcome of the septic patient [3]. Fortunately, the advent of “omic” technologies may allow for increased diagnostic support. The metabolomics approach offers the possibility to identify variations in fingerprint and metabolite profile that can be used to discriminate disease [4]. Metabolomics may become a promising tool for diagnosing newborn sepsis and monitoring therapy in the near future. Taking into consideration the studies carried out so far, it is reasonable to argue that the metabolomics technique can be considered an effective tool for the diagnosis of sepsis [5, 6]. While metabolomic studies are often encountered in human medicine for newborn sepsis [7–14], there is little knowledge related to the same approach for calf sepsis. The aim of the present study was to identify metabolomic biomarkers of sepsis in plasma by ^1^H-NMR spectroscopy to assess the severity and to predict outcomes.

## 2. Materials and Methods

### 2.1. Animals and Sepsis Criteria

Twenty newborn calves presented for treatment to the teaching hospital, 1–15 days of age with severe sepsis, and 10 clinically healthy age-matched control calves belonging to the faculty farm were used in this study. Inclusion criteria were the following: SIRS (systemic inflammatory response syndrome) criteria: body temperature > 39°C or <36°C, hearth rate < 100 or >160 beats/min, respiratory rate > 65/min or pCO_2_ > 50 mmHg, total leukocyte count > 12.000/mm^3^ or <4.000/mm^3^, or band neutrophil > 10%; sepsis criteria: at least two results above or more than SIRS + infection or infection suspected; and severe sepsis criteria: 4 sepsis results together with at least one organ failure (acute lung damage; coagulation abnormalities; thrombocytopenia; mental state changes; and liver, kidney, or/and cardiac insufficiency or acidosis due to hypoperfusion) [15].

### 2.2. Blood Sampling

Diseased calves were treated by antibiotics and fluids, and critical care was performed, when needed. Their blood samples were taken at set intervals (0, 3, 6, 12, 48, and 72 h) after admission to the animal hospital, and serum and plasma samples were harvested by centrifuging the blood at 1000 ×g for 15 min at 4°C.

### 2.3. Laboratory Analysis

Laboratory workup including complete blood count analysis (blood cell counts, hematocrit, hemoglobin, MCV, MCH, and MCHC), blood gas analysis (pH, pO_2_, pCO_2_, HCO_3_, base excess, and O_2_ saturation), and biochemical analyses (in serum samples: total protein, albumin, BUN, creatinine, lactate, AST, ALT, ALP, GGT, Na, and K) was performed by use of automated analyzers in the teaching hospital's clinical laboratory.

### 2.4. ^1^H-NMR Sample Preparation

NMR measurements were performed at the CERM/CIRMMP center of the ESFRI Instruct, University of Florence, in Florence, Italy. 350 *μ*L of each plasma sample was added to a total of 350 *μ*L of a phosphate sodium buffer (70 mM Na_2_HPO_4_; 20% (*v*/*v*) ^2^H_2_O; 0.025% (*v*/*v*) NaN_3_; 0.8% (*w*/*v*) sodium trimethylsilyl [2,2,3,3-2H4] propionate (TMSP), pH 7.4), and the mixture was homogenized by vortexing for 30 s. A total of 600 *μ*L of this mixture was transferred into a 5 mm NMR tube (Bruker BioSpin s.r.l.) for the analysis.

### 2.5. NMR Analysis

Monodimensional ^1^H-NMR spectra for all samples were acquired using a Bruker 600 MHz spectrometer (Bruker BioSpin s.r.l.; Rheinstetten, Germany) operating at 600.13 MHz proton Larmor frequency and equipped with a 5 mm PATXI 1H-13C-15N and 2H decoupling probe including a *z*-axis gradient coil, an automatic tuning and matching (ATM), and an automatic and refrigerated sample changer (SampleJet, Bruker BioSpin s.r.l.; Rheinstetten, Germany). A BTO 2000 thermocouple was served for temperature stabilization at the level of approximately 0.1 K at the sample. Before measurement, samples were kept for at least 5 minutes inside the NMR probehead, for temperature equilibration (310 K for plasma samples).

For each sample, three one-dimensional proton NMR spectra were acquired with different pulse sequences, allowing the selective detection of different molecular components [16].

- A standard nuclear Overhauser effect spectroscopy pulse sequence NOESY 1Dpresat (noesygppr1d.comp; Bruker BioSpin) pulse sequence, using 32 scans, 98,304 data points, a spectral width of 30.0459 Hz, an acquisition time of 2.7 s, a relaxation delay of 4 s, and a mixing time of 0.1 s, and with water peak suppression [17], was applied to obtain a spectrum which are visible signals of metabolites and high molecular weight macromolecules (lipids and lipoproteins).
- A standard spin echo Carr-Purcell-Meiboom-Gill (CPMG) [18] (cpmgpr1d.comp; Bruker BioSpin) pulse sequence applied to a standard 1D sequence, with 32 scans, 73,728 data points, a spectral width of 12,019 Hz, and a relaxation delay of 4 s, was used for the selective observation of low molecular weight metabolites, suppressing signals arising from macromolecules.
- A standard diffusion-edited [19] (ledbgppr2s1d.comp; Bruker BioSpin) pulse sequence, using 32 scans, 98,304 data points, a spectral width of 18,028 Hz, and a relaxation delay of 4 s, was applied to suppress metabolite signals.

The acquisition conditions used in this work are perfectly in line with what is validated and recommended in literature [20]. These conditions are suggested by Bruker (the NMR machine vendor) for metabolomic analysis [21].

### 2.6. Spectral Processing

Free induction decays were multiplied by an exponential function equivalent to a 0.3 Hz line-broadening factor before applying Fourier transform.

Transformed spectra were automatically corrected for phase and baseline distortions and calibrated to anomeric glucose doublet at 5.24 ppm using TopSpin 3.2 (Bruker BioSpin s.r.l.).

Each 1D spectrum in the range between 0.2 and 10.00 ppm was segmented into 0.02 ppm chemical shift bins, and the corresponding spectral areas were integrated using AMIX software (version 3.8.4, Bruker BioSpin). Binning is a means to reduce numbers of total variables and to compensate for small shifts in the signals, making the analysis more robust and reproducible [22]. Regions containing residual water signal were removed.

### 2.7. Statistical Analysis

For laboratory data, normality test was performed to explain if all data are parametric or nonparametric. For comparing the parametric values, ANOVA and Tukey tests were performed and calculated as mean ± SD. For comparing the nonparametric ones, Mann–Whitney *U* test was performed and presented as median (SPSS 21.0). Statistical significance was evaluated as *P* value < 0.05.

Principal component analysis (PCA) was used as a first exploratory analysis. Orthogonal projections to latent structures (OPLS) was chosen as supervised technique. OPLS is a multivariate projection method which is frequently applied for modelling spectroscopic data. This algorithm is able to separate “response-related” and “response-orthogonal” variation in data, providing benefits in terms of model interpretation compared to PCA or to PLS [23]. All the accuracies reported and the confusion matrix for different classifications were assessed by means of 100 cycles of a Monte Carlo cross-validation scheme (MCCV, R script in-house developed). In this case, 90% of the data were randomly chosen at each iteration as a training set to build the model. Then the remaining 10% was tested and sensitivity, specificity, and accuracy for the classification were assessed. The procedure was repeated 100 times to derive an average discrimination accuracy for each group of subjects.

For metabolomic data, twenty-two metabolites, corresponding to well-defined and resolved peaks in the spectra, were assigned. Signal identification was performed using a library of NMR spectra of pure organic compounds, public databases (e.g., HMBD), and literature data.

The molecule 1,4-dioxane was used as reference standard to perform a quantitative NMR (qNMR) analysis on the plasma samples to obtain absolute concentration (*μ*M) values of the metabolites analyzed. The concentrations of the various metabolites in the different spectra were calculated by spectral fitting and integration of the signal area using in-house MATLAB® scripts and the concentrations of peak integrals are compared to the internal standard peak integral to obtain absolute concentrations, using cpmg spectra. The use of CPMG spectra for quantitative analysis is well supported by literature [24]. Kruskal-Wallis test followed by Dunn post hoc analysis was chosen to infer metabolite differences among the groups on the biological assumption that metabolite concentrations are not normally distributed. False discovery rate correction was applied using the Benjamini-Hochberg method (FDR) and adjusted *P* value < 0.05 was considered statistically significant [25]. MetaboAnalyst 3.0 was used for pathway analysis [26].

## 3. Results

### 3.1. Clinical and Laboratory Data

The diseased calves were recumbent, weak, dehydrated (≥8%), and hypothermic (*T* = 36.8°C). Poor pulse quality and occasional pulse deficits were revealed by palpation and auscultation, and heart rate (92 beats/min) was within normal limits except two animals in which atrial standstill was observed. The animals had metabolic acidosis, hyperkalemia, lactatemia, and leukocytosis. O2 saturation remained decreased, and BUN, creatinine, and AST increased at set intervals in spite of therapy (Supplemental Tables 1, 2 and 3). Three animals died at the 24th hour and at the 48th hour, and two animals at the 72nd hour. The remaining animals (11) died after the 72nd hour.

### 3.2. NMR-Based Metabolites

**Figure 1:**
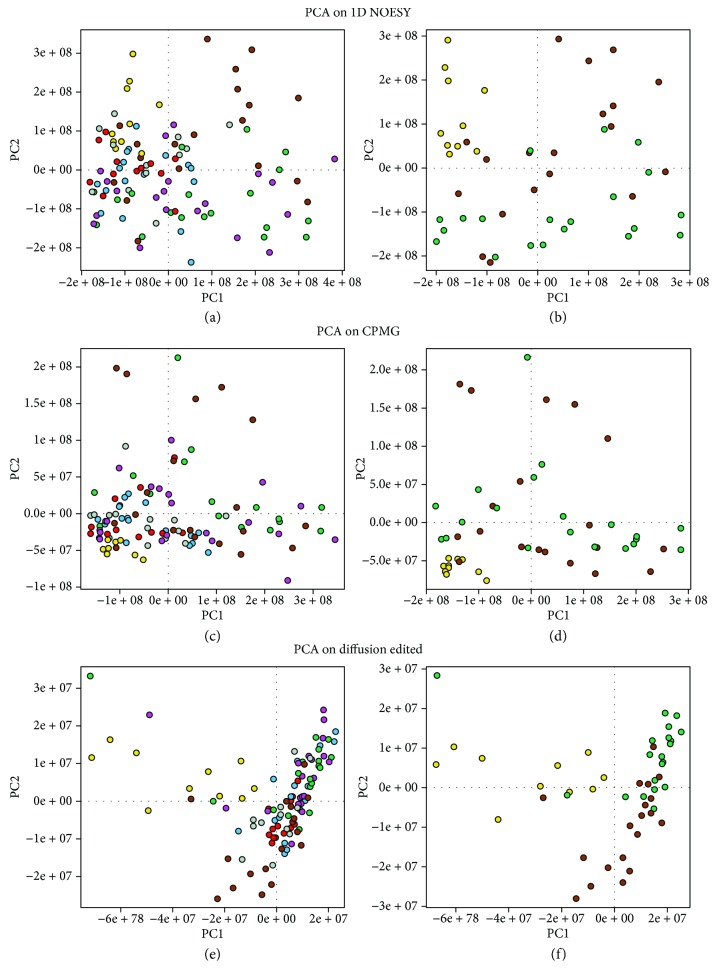
Principal component analysis (PCA) score plots. Each dot represents a single NMR spectrum of plasma samples collected at different time points: healthy animals (Ha, yellow dots), 0 hour (brown dots), 3rd hour (green dots), 6th hour (magenta dots), 24th hour (light-blue dots), 48th hour (gray dots), and 72nd hour (red dots). (a) PCA on all 1D NOESY spectra; (b) PCA on Ha, 0 h, and 3rd-hour 1D NOESY spectra; (c) PCA on all CPMG spectra; (d) PCA on Ha, 0 h, and 3rd-hour CPMG spectra; (e) PCA on all diffusion-edited spectra; (f) PCA on Ha, 0 h, and 3rd-hour diffusion-edited spectra.

NMR spectra of all 113 plasma samples were acquired, sample n°15 (0 h) was removed from our analysis because the spectrum was of bad quality. Processed plasma samples of newborn calves, collected at different time points (Healthy animals (Ha); diseased animals at 0 hour; diseased animals at the 3rd hour; diseased animals at the 6th hour; diseased animals at the 24th hour; diseased animals at the 48th hour; and diseased animals at the 72nd hour), have been analyzed firstly using the unsupervised multivariate method (PCA) to gain an overview on the main changes responsible for sepsis.  Figure 1 shows the PCA score plots on 1D NOESY, CPMG, and diffusion-edited spectra of plasma samples. Unsupervised principal component analysis of all plasma spectra is sufficient to discriminate, in all the three experiments, healthy animals (Ha, yellow dots) from calves with sepsis, and particularly 0 h blood samples (brown dots) can be distinguished from the other time points, suggesting that from the 3rd to the 72nd hour, plasma profile/metabolism of newborn calves with sepsis is very similar.

**Figure 2:**
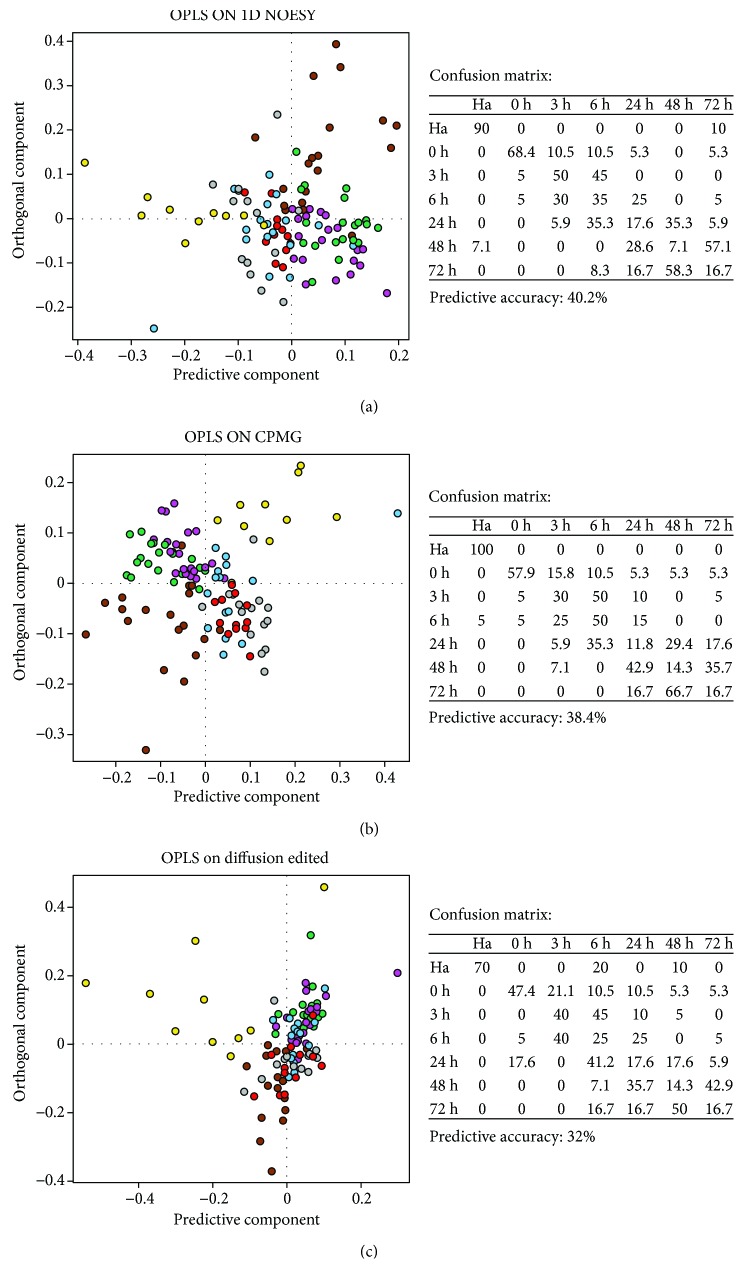
OPLS score plot of all plasma samples: healthy animals (Ha, yellow dots), 0 hour (brown dots), 3rd hour (green dots), 6th hour (magenta dots), 24th hour (light-blue dots), 48th hour (gray dots), and 72nd hour (red dots). (a) NOESY experiment; (b) CPMG experiment; (c) diffusion-edited experiment. Confusion matrices and related prediction accuracy of cross-validation analyses are reported for each model.

Subsequently, the OPLS-supervised method was employed to extract and visualize latent and hidden variation characteristics of sepsis progression. OPLS models were built on NOESY, CPMG, and diffusion-edited experiments, respectively (Figures 2(a)–2(c)) and the first 10 components were retained in the model. All the three models, as shown by cross-validations (Figure 2), are able to excellently identify healthy animals (Ha) with 90% and 100% accuracy using OPLS models built on NOESY and CPMG experiments, respectively. Interestingly, 0 h plasma samples appear the most discriminated, while from the 3rd hour to the 72nd hour, each group cannot be recognized with good accuracy. Using the same statistical approach, discrimination analysis was also attempted to check whether the metabolic profile at time 0 differed between animals that died early from those that died later to check if the metabolic profile can predict animal outcome before starting the therapeutic treatment. However, the predictive accuracies obtained from the models were very low (predictive accuracy on the NOESY model: 39%, predictive accuracy on the CPMG model: 44%, and predictive accuracy on the diffusion-edited model: 38%); this unsatisfactory result can be ascribed to the fact that the number of samples per group is too low or also because all animals (except one) died after 72 hours leading to the conclusion that all individuals share the same pathological fingerprint.

**Figure 3:**
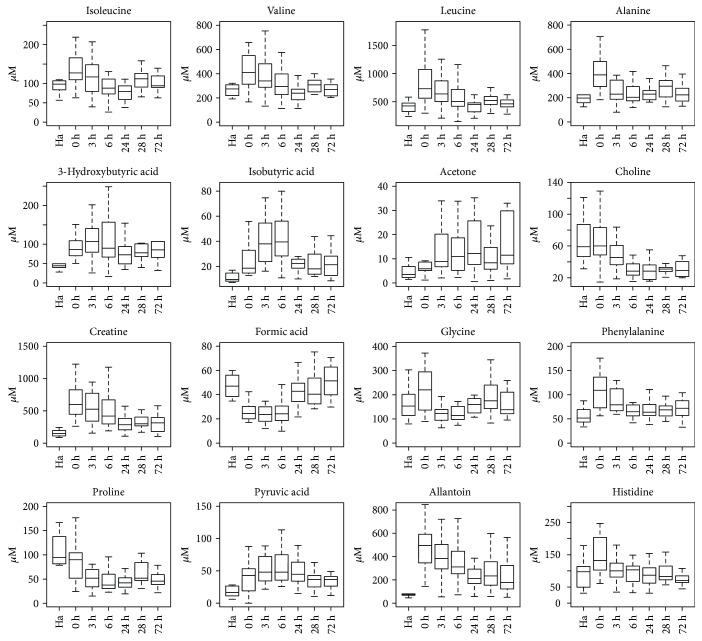
Box plots of the metabolites differentially concentrated in the seven calf groups. Ha: healthy animals; 0 h: diseased animals at 0 hour; 3 h: diseased animals at the 3rd hour; 6 h: diseased animals at the 6th hour; 24 h: diseased animals at the 24th hour; 48 h: diseased animals at the 48th hour; 72 h: diseased animals at the 72nd hour.

NMR spectra were also analyzed to identify which metabolites are altered in the seven groups of calves. The twenty-two identified and quantified metabolites are listed in Table 1; *P* values are reported only for metabolites that differ significantly (*P* value < 0.05) (Figure 3). Diseased samples at 0 hour compared with healthy animals appeared to be richer in isoleucine, leucine, valine, alanine, creatine, phosphocreatine, glycine, phenylalanine, and histidine which decreased gradually along hours. Allantoin, 3-hydroxybutyric acid, acetone, and isobutyric acid remained increased at all the hours. While acetone gradually increased, choline and proline gradually reduced along hours. Formic acid is decreased at 0 hour then increased gradually.

## 4. Discussion

NMR-based metabolomics were evaluated at set intervals, for the first time, in newborn calves with severe sepsis. The data indicated that metabolomics was a feasible tool for the identification of septic plasma profiles and quantification of potential meaningful biomarkers for severe septic newborn calves. Newborn sepsis commonly originates from an abrupt evolution of infections in the first 4 weeks of life. Clinical signs of newborn sepsis are often nonspecific, subtle, and inconspicuous and therefore demand a high index of suspicion for early diagnosis [2]. In this present study, clinical and laboratory results at admission were in accordance with most references. Clinical findings especially mental status contrary to leukocytosis, metabolic acidosis, lactatemia, and hyperkalemia could not be corrected properly. In the diseased animals, O_2_ saturation remained decreased; however BUN, creatinine, and AST were increased along all hours. All the animals (except one) died after 72 h. This may be attributed to multiple-organ failure. These laboratory results on animal serum samples obtained by analytical methods optimized for human matrix may be questionable, even though they may be considered indicative.

There are a variety of tests that are helpful for screening newborns with sepsis. Traditional biomarkers do not sufficiently discriminate between sepsis and SIRS. Thus, the identification of more sensitive reliable and rapidly measured biomarkers to differentiate sepsis from SIRS and monitor disease progression and treatment efficacy is a matter of intense interest. This study has shown that NMR metabolomics could represent an optimal tool for faster identification of newborn calves with severe sepsis and a complementary tool to classical methods used until now to stratify better the prognosis in septic shock and severe sepsis. The usefulness of metabolomics in exploring underlying biochemical mechanisms of sepsis could—after validation—provide novel candidate mechanisms to confirm or follow-up the progression of newborn early- or late-onset sepsis [27, 28]. Urinary ^1^H-NMR and GC-MS metabolomics predict early- and late-onset newborn sepsis [8]. In the study, the variables significantly contributing to the separation of the septic samples from healthy animals in multivariate analysis included several metabolites such as acetate, glycine, glucose, acetone, lactate, and lysine, whereas control samples were characterized by citrate and creatinine. In another ^1^H-NMR human study, several metabolites (maltose, glucose, biotine, methylamine, inosine, methylguanidine, creatine, myoinositol, and quinolinic acid) were found significantly changed in septic neonates at the onset of the disease [28]. In this current study, identified and quantified metabolites and their functions are the following: *valine*: synthesis of glutamine and alanine and balance among branched-chain amino acid (BCAA); *proline*: collagen structure and function, neurological function, and osmoprotection; *choline*: synthesis of betaine, acetylcholine, phosphatidylcholine, and sarcosine; *alanine*: inhibition of pyruvate kinase and hepatic autophagy, gluconeogenesis, transamination, and glucose-alanine cycle; *phenylalanine*: activation of BH4 (a cofactor for NOS) synthesis, synthesis of tyrosine, and neurological development and function; *leucine*: regulation of protein turnover through cellular mechanistic target of rapamycin signaling and gene expression, activator of glutamate dehydrogenase, BCAA balance, and flavor enhancer; *isoleucine*: synthesis of glutamine and alanine and balance among BCAA; *histidine*: protein methylation, hemoglobin structure and function, antioxidative dipeptides, and one-carbon unit metabolism; *glycine*: calcium influx through a glycine-gated channel in the cell membrane, purine and serine syntheses, synthesis of porphyrins, inhibitory neurotransmitter in the central nervous system, and coagonist with glutamate for N-methyl-D-aspartate receptors; and *creatine*: antioxidant, antiviral, antitumor, energy metabolism in muscle and brain, and neurological and muscular development and function [29]. Most metabolites increased at 0 hour (Supplemental Table 1) indicated the response to metabolic deficits for early-onset sepsis. These metabolites gradually decreased at other hours following the therapy. Also, the presence of ketone bodies such as acetone, 3-hydroxybutyric acid, and isobutyric acid in the plasma of the septic group at all hours suggests a compensatory reaction to a reduced level of ATP [8]. A simplified list of the most contributing metabolic pathways is reported in Supplemental Table 4 based on MetaboAnalyst software. The analysis showed alteration in biochemical pathways like aminoacyl-tRNA biosynthesis (histidine, phenylalanine, glycine, valine, alanine, isoleucine, leucine, and proline), valine/leucine/isoleucine biosynthesis (pyruvate, leucine, valine, and isoleucine), nitrogen metabolism (phenylalanine, histidine, glycine, and formate), glycine/serine and threonine metabolism (choline, glycine, creatine, and pyruvate), and synthesis and degradation of ketone bodies (3-hydroxybutyrate, acetone). The analysis was calculated based on the significance value (*P* value < 0.05) of the pathway enrichment analysis. In our previous study [30] where the measurements were not at set intervals, the whole lipid soluble metabolites such as sphingomyelin and fatty acids including PUFA were reduced and attributed to a great systemic energy deficit during sepsis. Being in accordance with Langley and Wong [31], it is said that metabolomic changes in sepsis suggest an energy crisis in nonsurvivors. Some metabolites such as formic acid in this present study were similar to those in the previous study. Remarkable increased allantoin of the septic group in this present study can be an indication of microbial overgrowth or oxidative stress. Cell death from cytochrome oxidase inhibition by formic acid believed to result partly from depletion of ATP, reducing energy concentrations so that essential cell functions cannot be maintained. In this regard, gradually increasing of formic acid being decreased at 0 hour can be meaningful for prognosis of septic calves.

## 5. Conclusions

To our knowledge, this is the first study where the measurements were at set intervals showing that ^1^HNMR-based metabolomics approach may indeed be a powerful instrument providing knowledge about the factors responsible of the metabolic modifications in newborn septic calves. NMR metabolomics proved to be an optimal tool for faster identification of newborn calves with sepsis using plasma samples. Although identified and quantified metabolites may become potential biomarkers for diagnosing newborn sepsis and monitoring therapy of sepsis in calves, the extent of the predictive and prognostic values of this given set of metabolites will be required for clinical trials.

## Figures and Tables

**Table 1:** Concentrations in *μ*M (mean ± SD) of the metabolites assigned in plasma samples. Significantly different *P* values from the comparisons are also reported.

Metabolites (*μ*M)	Healthy animals (*n* = 10)	Diseased animals (hours)						*P* value
		0 (*n* = 19)	3rd (*n* = 20)	6th (*n* = 20)	24th (*n* = 17)	48th (*n* = 14)	72nd (*n* = 12)	
Isoleucine	98.8 ± 28.3	147.8 ± 67	122.3 ± 57.4	98.5 ± 53	82.3 ± 36.4	111.5 ± 27.5	106.1 ± 36.5	=0.01 (0 h versus 6 h) <0.001 (0 h versus 24 h) <0.05 (3 h versus 24 h)

Valine	227.3 ± 76.2	434.7 ± 182.7	380.6 ± 157	323.6 ± 144.5	232.9 ± 80.6	304.6 ± 56.8	284.9 ± 100.1	<0.05 (Ha versus 0 h) <0.001 (0 h versus 24 h) =0.005 (0 h versus 24 h) <0.05 (0 h versus 72 h)

Leucine	405.7 ± 111.2	851.5 ± 421.8	701 ± 355	586.4 ± 303.6	425 ± 145.3	538.9 ± 144.2	483.6 ± 137.9	=0.001 (Ha versus 0 h) <0.05 (Ha versus 3 h) <0.001 (0 h versus 24 h) <0.05 (3 h versus 24 h) <0.05 (0 h versus 72 h)

Alanine	236.9 ± 142.3	398.1 ± 154.2	341.8 ± 333.4	274.6 ± 191.6	226.2 ± 51.5	293 ± 123.2	242.2 ± 97	<0.05 (Ha versus 0 h) <0.05 (0 h versus 3 h) <0.05 (0 h versus 6 h) <0.05 (0 h versus 24 h) <0.05 (0 h versus 72 h)

3-Hydroxybutyric acid	61.4 ± 44.6	94.2 ± 38.9	129.8 ± 105	134 ± 146	84.4 ± 44.2	111.2 ± 118.2	124.7 ± 132	<0.05 (Ha versus 3 h)

Isobutyric acid	10.9 ± 3.7	27.1 ± 16.14	40 ± 17.8	41.2 ± 17.9	22.7 ± 8.5	21.6 ± 9.8	21.6 ± 10.6	<0.05 (Ha versus 0 h) <0.00001 (Ha versus 3 h) <0.00001 (Ha versus 6 h) <0.05 (Ha versus 24 h) <0.05 (Ha versus 48 h) <0.05 (Ha versus 72 h) <0.05 (0 h versus 3 h) <0.05 (0 h versus 6 h) <0.05 (3 h versus 24 h) <0.05 (3 h versus 48 h) <0.05 (3 h versus 72 h) <0.05 (6 h versus 24 h) <0.05 (6 h versus 48 h) <0.05 (6 h versus 72 h)

Acetic acid	54 ± 17	234.24 ± 536	110.31 ± 54.44	86.9 ± 30.87	116.7 ± 115.5	77.5 ± 62.3	67.6 ± 43.7	

Acetone	6.7 ± 8.6	10 ± 9.6	14.6 ± 11.8	15.4 ± 14.6	15.4 ± 11.4	16.6 ± 25.5	26.3 ± 33.7	=0.05 (Ha versus 3 h) =0.05 (Ha versus 6 h) =0.05 (Ha versus 24 h) =0.05 (Ha versus 72 h)

Choline	68.5 ± 29.3	66.8 ± 29.5	47.5 ± 18.2	32 ± 13.5	29.6 ± 11.7	32.7 ± 12.3	33 ± 14.6	<0.001 (Ha versus 6 h) <0.001 (Ha versus 24 h) <0.05 (Ha versus 48 h) <0.05 (Ha versus 72 h) <0.001 (0 h versus 6 h) <0.001 (0 h versus 24 h) <0.001 (0 h versus 48 h) <0.001 (0 h versus 72 h) <0.05 (3 h versus 6 h) <0.05 (3 h versus 24 h) <0.05 (3 h versus 48 h) <0.05 (3 h versus 72 h)

Creatine	168.8 ± 74.5	702.9 ± 397	628.4 ± 431.3	533.5 ± 401.6	309 ± 144	336 ± 103.4	305 ± 146.3	<0.00001 (Ha versus 0 h) <0.001 (Ha versus 3 h) <0.001 (Ha versus 6 h) <0.05 (Ha versus 48 h) <0.001 (0 h versus 24 h) <0.05 (0 h versus 48 h) =0.001 (0 h versus 72 h) <0.05 (6 h versus 24 h) <0.05 (3 h versus 72 h)

Phosphocreatine + creatinine	108 ± 30.8	399.3 ± 153.5	378.4 ± 226.8	333.2 ± 221	219 ± 111.2	219 ± 75.5	207.8 ± 95.7	<0.00001 (Ha versus 0 h) =0.00001 (Ha versus 3 h) <0.001 (Ha versus 6 h) <0.05 (Ha versus 24 h) <0.05 (Ha versus 48 h) <0.05 (Ha versus 72 h) <0.05 (0 h versus 24 h) <0.05 (0 h versus 48 h) <0.05 (0 h versus 72 h) <0.05 (3 h versus 24 h) <0.05 (3 h versus 48 h) <0.05 (03 versus 72 h)

Formic acid	51 ± 17.8	27.3 ± 10.4	24.6 ± 9.3	24.7 ± 9.2	43.4 ± 16	44.5 ± 14.3	54.3 ± 22.3	<0.001 (Ha versus 0 h) <0.001 (Ha versus 6 h) <0.001 (Ha versus 24 h) <0.05 (0 h versus 24 h) <0.05 (0 h versus 48 h) <0.001 (0 h versus 72 h) <0.001 (3 h versus 24 h) <0.001 (3 h versus 48 h) <0.001 (3 h versus 72 h) <0.001 (6 h versus 24 h) <0.001 (6 h versus 48 h) <0.001 (6 h versus 72 h)
D-Glucose	1400 ± 245.5	1811 ± 1196	1351 ± 709.5	1816 ± 1226.4	1697 ± 652	1783 ± 439	1714.4 ± 1423	

Glycine	171.2 ± 76.1	223.8 ± 90.5	160.3 ± 137	142.2 ± 86	176.3 ± 68.2	198 ± 73.2	170 ± 77.25	<0.05 (0 h versus 3 h) <0.05 (0 h versus 6 h) <0.05 (3 h versus 48 h) <0.05 (6 h versus 48 h)

Phenylalanine	57.2 ± 16.7	112.2 ± 52.7	95.2 ± 50	77.6 ± 44.8	83 ± 66.5	71.3 ± 17.9	71.6 ± 21	<0.05 (Ha versus 0 h) <0.05 (Ha versus 3 h) <0.05 (0 h versus 6 h) <0.05 (0 h versus 24 h) <0.05 (3 h versus 6 h)

Proline	108.7 ± 34.2	84 ± 38.8	92.8 ± 14.5	71.2 ± 21.3	43 ± 14.5	63.3 ± 32.2	48 ± 16.6	<0.05 (Ha versus 3 h) <0.05 (Ha versus 6 h) <0.05 (Ha versus 24 h) <0.05 (Ha versus 48 h) <0.05 (Ha versus 72 h) <0.05 (0 h versus 3 h) <0.05 (0 h versus 6 h) <0.05 (0 h versus 72 h)

Pyruvic acid	21.8 ± 16.7	49.6 ± 56	51.2 ± 21.3	56 ± 26.8	48.8 ± 22.6	42.4 ± 24.4	39.5 ± 24.3	<0.05 (Ha versus 3 h) <0.05 (Ha versus 6 h) <0.05 (Ha versus 24 h)

Tyrosine	44.64 ± 23.9	53.39 ± 16.64	50.74 ± 27.90	41.22 ± 17.07	36.54 ± 14.87	35.89 ± 12.76	34.62 ± 14.93	

Dimethyl sulfone	7.91 ± 1.94	15.06 ± 8.74	14.11 ± 9.1	13.19 ± 9.37	11.91 ± 6.63	11.15 ± 4.93	10.54 ± 4.87	

Allantoin	71.8 ± 35.05	501.4 ± 238.9	423.4 ± 213	376.4 ± 200.2	256.8 ± 169.2	268.3 ± 155.8	238.7 ± 165.7	<0.00001 (Ha versus 0 h) <0.00001 (Ha versus 3 h) <0.001 (Ha versus 6 h) <0.05 (Ha versus 24 h) <0.05 (Ha versus 48 h) <0.05 (Ha versus 72 h) <0.05 (0 h versus 24 h) <0.05 (0 h versus 48 h) <0.05 (0 h versus 72 h) <0.05 (3 h versus 24 h) <0.05 (3 h versus 72 h)

GPC	32.9 ± 55.8	44.7 ± 27	34.9 ± 27.4	27.6 ± 23.4	25 ± 17.8	25.5 ± 25.4	28.8 ± 29.9	

Histidine	98.6 ± 50.6	151.5 ± 62.2	120.3 ± 86.9	110.7 ± 68.9	87.4 ± 32.9	94.8 ± 30.3	78.8 ± 32.5	<0.05 (0 h versus 24 h) <0.05 (0 h versus 72 h)

## Supplementary Materials

Supplementary Materials Supplemental Table 1: blood gas analysis. Supplemental Table 2: complete blood count analysis. Supplemental Table 3: biochemical profile. Supplemental Table 4: an integrated analysis based on MetaboAnalyst software: view of contributing pathways.
