# Supplementary material for: NMR-Based Plasma Metabolomics at Set Intervals in Newborn Dairy Calves with Severe Sepsis
Source: Mediators Inflamm. 2018 Mar 21;2018:8016510. doi: 10.1155/2018/8016510 (PMC5883973; doi:10.1155/2018/8016510)
Supplement: Supplementary Materials — Supplemental Table 1: blood gas analysis. Supplemental Table 2: complete blood count analysis. Supplemental Table 3: biochemical profile. Supplemental Table 4: an integrated analysis based on MetaboAnalyst software: view of contributing pathways. [file 8016510.f1.pdf]

Supplemental Table 1 Blood gas analysis.

Parameters	Healthy animals	Diseased animals (Hours)						P value
	(n=10)	0 (n:20)	3. (n:20)	6 (n:20)	24 (n:17)	48 (n:14)	72 (n:12)	
Ph	7.38±0.00a	7.00±0.03b	7.27±0.7b	7.28±0.01a	7.29±0.01a	7.28±0.02a	7.31±0.02a	<0.001
PCO_2, mmHg	44.20±1.10	51.50±2.59	47.85±7.59	47.35±1.28	51.64±1.65	49.64±1.31	50.41±2.49	
PO_2, mmHg	32.50±0.6a	26.35±1.9ab	31.55±9.18ab	26.75±2.25ab	24.05±1.48b	23.07±1.78b	22.25±1.89b	0.29
$HCO_{3,}$ mol/L	27.99±1.37a	13.61±1.34c	7.27±0.7b	22.66±0.99b	25±0.90ab	23.67±1.22ab	25.60±1.30ab	<0.001
BE, mol/L	4.14±0.61a	-17.36±1.74c	47.85±7.59	-4.02±1.13b	-1.92±1.01b	-3.23±1.47b	-0.95±1.44ab	<0.001
O_2 saturation, %	70.30±1.24a	24.05±4.27b	48.70±19.18b	39.75±4.70b	35.29±3.97b	32.57±4.30b	32.25±4.27b	<0.001

Supplemental Table 2 Complete blood count analysis.

Parameters	Healthy animals	Diseased animals (Hours)						P value
	(n=10)	0 (n:20)	3. (n:20)	6 (n:20)	24 (n:17)	48 (n:14)	72 (n:12)	
WBCs, $10^3/\mu L$	8.94d	17.82a	13.13±8.79ab	13.99ab	14.69a	11.72bc	11.18bcd	<0.003
Lymphocytes, $10^3/\mu L$	2,34ab	3,70a	3.79a	2,30b	2,80ab	2,99ab	3,59ab	>0.05
Monocytes, $10^3/\mu L$	0.34±0.03b	0.61±0.07a	0.40±0.15ab	0.39±0.04ab	0.53±0.05ab	0.43±0.06ab	0.54±0.06ab	>0.05
Granulocytes, $10^3/\mu L$	2.66±017c	15.75±2.13a	8.93±7.29ab	11.65±1.81ab	11.94±1.36ab	8.19±1.25bc	8.26±1.52bc	<0.015
RBCs, $10^6/\mu L$	8.00±0.40ab	10.12±0.50a	7.44±1.95b	7.72±0.47b	8.59±0.51ab	8.45±0.49ab	8.19±0.50ab	<0.008
MCV, fl	33.36±1.21b	38.16±0.80a	37.48±3.58a	37.60±0.82a	37.55±0.85a	36.92±0.76a	37.20±0.90a	<0.020
PCV, %	27.03±2.16b	38.86±2.34a	28.14±8.88b	29.33±2.14ab	32.50±2.32ab	31.27±2.08ab	30.50±2.19ab	<0.009
MCH, pg	12.62±0.43	12.83±0.25	12.53±1.21	13.00±0.41	12.41±0.37	12.70±0.28	12.42±0.24	>0.05
MCHC, g/dl	38.08±0.35a	33.86±0.54b	33.68±2.74b	34.74±0.65b	33.38±0.99b	34.65±0.78b	33.66±0.72b	<0.003
Hb, g/dl	10.29±0.81	13.12±0.76	9.43±2.84b	10.17±0.73	10.78±0.74	10.76±0.70	10.21±0.68	<0.031

Supplemental Table 3 Biochemical profile.

Parameters	Healthy animals	Diseased animals (Hours)						P value
	(n=10)	0 (n:20)	3 (n:20)	6 (n:20)	24 (n:17)	48 (n:14)	72 (n:12)	
BUN, mg/dL	8.90±1.37b	54.55±5.92a	51.80±27.44a	48.25±4.93a	45.29±4.46a	44.64±5.99a	48.25±8.18a	<0.001
Creatinine, g/dL	1.14±0.08b	3.99±0.57a	3.74±2.30a	3.50±0.52a	2.82±0.53ab	2.57±0.41ab	2.54±0.49ab	0.014
AST, IU/L	48.50±3.42b	134.4±19.9ab	152.8±111.4ab	195.3±24.8a	242.6±30.1a	208±30.59a	207.8±25.6a	<0.001
ALT, IU/L	12,50c	38,00b	29.5b	39,50ab	76,00a	80,5a	75,50a	0.002
ALP, IU/L	484±68.51	609.2±74.1	450±257	488.4±57.01	475.5±54.8	444.4±40.4	403.6±39.9	
GGT, IU/L	62,50a	66,00a	46a	44,00a	54,00a	101,00a	54,00a	
Protein, g/dL	6.16±0.23 a	5.64±0.37 a	4.01±0.82c	4.24±0.21b	4.93±0.25ab	5.25±0.29ab	5.09±0.22 ab	0.001
Albumin, /dL	3.46±0.10 ab	3.58±0.14a	2.59±0.37c	2.72±0.08c	3.07±0.09bc	3.25±0.10ab	3.27±0.10ab	<0.001
Na, mmol/L	146.20±0.92c	142.40±1.97c	150.7±7.94bc	151.8±1.5bc	160±2.22ab	160.4±2.6ab	162.2±4.3a	<0.001
K, mmol/L	3.95±0.10b	6.25±0.31a	5.24±1.14ab	4.51±0.25b	4.20±0.26b	4.49±0.29b	4.75±0.23b	<0.001
Lactate, mmol/L	1.16±0.15b	5.31±0.99a	6.78±5.13a	5.95±1.06a	3.31±0.58ab	2.77±0.54ab	2.70±0.68ab	0.002

Supplemental Table 4 An integrated analysis based on Metaboanalyst software: view of contributing pathways.

Pathway Name	*P*-value	Holm *P*-value	FDR
Aminoacyl-tRNA biosynthesis	6.4289×10^{-9}	5.1431×10^{-7}	5.1431×10^{-7}
Valine, leucine and isoleucine biosynthesis	2.0877×10^{-5}	0.0016493	8.3509×10^{-4}
Nitrogen metabolism	9.3233×10^{-5}	0.0072722	0.0024862
Glycine, serine and threonine metabolism	2.127×10^{-4}	0.016378	0.0042541
Synthesis and degradation of ketone bodies	6.1204×10^{-4}	0.0097926	0.0097926
